# Progress in Medicine

**Published:** 1898-02-05

**Authors:** 


### PROGRESS IN MEDICINE.

### RENAL MEDICINE.

**Urology.**—Schultess[1] has investigated the relation of albumosuria to fevers, and finds that 75 per cent. of the recorded cases, where it was present, were suffering from fevers of various kinds. Next he examined 56 patients with non-febrile diseases, and detected albumose in 23 per cent. only, while in 59 cases of fever he found it present in 90 per cent. The albumose in the acute fevers corresponded pretty closely with the curve of the fever. Thus it is in fever four times as frequent as in other morbid states. In his experiments he excluded urines containing albumen, removed the nucleo-albumens by acetic acid, and then added to the filtrate five or six volumes of absolute alcohol. This was allowed to stand 24 hours, and was filtered. Finally, the filtrate was dissolved in warm water, and the biuret reaction employed. On the other hand, Hugouneng[2] refers albumosuria to cases of bone disease and syphilis, and notices that the substance when precipitated by sulphate of ammonia is redissolved on adding water, while true albumen remains insoluble. He also refers to Byrom Bramwell's case, where an average of 45 grammes daily was excreted and deposited in a crystalline form. Bradford[3] regards albumosuria as of little importance, except as symptom of fever and of pus producing microbes; or when occurring in granular kidney, and possibly in the course of other forms of Bright's disease; or, lastly, as a symptom of ovarian disease. In granular kidney and ovarian disease large amounts may be present, though the cause is totally unknown. Generally, however, it is associated with febrile states, and its appearance with granular kidney and ovarian disease is rare and exceptional. Not infrequently true albumen follows the excretion of albumose, from its irritating effect on the kidneys, and this is said to be the explanation of the temporary albuminuria of many fevers. For a time, indeed, the two substances may be excreted together.

Recently Dieulafoy[4] has revived the discussions upon hæmaturia without an apparent disease; but he has to confess that, important as these cases are, and difficult as they are to diagnose, their origin is unknown. A patient of his own, aged 18, suffered daily for two years when he walked or even stood for a long while. There were no other symptoms except anæmia, and under the use of turpentine he was cured in ten weeks, and remained well five years after, though he still took turpentine daily as a precaution. In another patient, after six months' hæmorrhage, as the right kidney was tender and gave pain, it was explored, but no cause was found. After the operation the hæmorrhage ceased, and the woman has remained in good health. Such hæmorrhages have been ascribed to congestion, hæmophilia, and purpura; and the difficulty is usually to exclude tuberculosis, cancer, and calculus, which are certainly absent in many well-attested instances. Profuse hæmorrhages, it must be remembered, occasionally occur in granular kidney disease, just as epistaxis is found in the same condition. The detection of blood is not always easy. One part in 15,000 is enough to cause smokiness (W. L. Otis), and one in 500 gives a cherry red. Dongany[5] improves the ordinary spectroscopic examination for it by adding ammonium sulphide to the acidified urine, which converts the hæmoglobin into hæmochromogen, and gives a more intense spectrum. He recommends also the following simple test: To 10 c.c. of urine are added 1 c.c. of ammonium sulphide, and the same amount of pyridin. The fluid instantly becomes of an orange-red colour, more or less intense with the quantity of blood present. If vomit, sputa, or fæcal matter have to be tested they should be first dissolved in caustic soda, and the other tests then added. He finds that Heller's test (by boiling urine and liquor potassæ, which gives a red precipitate if much blood is present) is useful and simple, but is affected by other substances as well as blood, and requires the presence of nearly one part of blood in a thousand.

Hæmatoporphyrin is one of the four normal pigments of the urine. The others, according to Rose Bradford,[6] are urochrome, urobilin, and uroerythrin. In health it is only found in traces. Its presence in pathological states is probably due to some internal hæmorrhage and destruction of blood corpuscles, such as precedes the appearance of urobilin. Nakarai[7] found it, however, in only 10 out of 144 cases. Six of these were due to lead poisoning, one to sulphonal, one to rheumatism, and two to intestinal tuberculosis with bleeding from the bowels. The quantity in plumbism was usually greater after the attack of colic, and decreased with convalescence, but the writer does not believe the presence of the pigment has any prognostic value, nor is the fact certain that it depends upon some intestinal hæmorrhage. Indeed, the carnivora might be supposed to excrete it if that were the case, from the blood elements in the flesh passing down their intestinal canals. Bradford discusses in the same paper the importance of the allied pigment urobilin. When present in abnormal quantities the urine is of a brownish-red colour, and the patient has a yellowish tinge, so that the case is often mistaken for true jaundice. However, the bile tests in such conditions do not give complete reactions. The green colour with nitric acid is absent from the series, and the spectrum shows the urobilin band, while bile pigment does not. In short, we have here an indication of the breaking up of blood pigment in the body. Typical instances are seen in hæmoglobinuria, in pernicious anæmia, in pyæmia, in post-operative bleeding within the body, as well as in purpura and many febrile diseases. The lemon-coloured tint of the patient, the dark-coloured urine, and the absence of the complete reaction for bile, should lead us in such cases to suspect some important destruction of blood corpuscles. Hæmaturia is sometimes produced by an excess of oxalic acid in the food, as in a case lately recorded by W. T. McCardie.[8] The patient was a healthy man of 45, who ate little meat, but partook largely of sugar and starchy food, with much tea and vegetables, especially rhubarb and spinach. He complained of lumbar pain, not worse after exertion, and passed at times very acid urine, containing blood albumen and 2·8 per cent. of urea with many octohedral crystals of oxalate of lime. When the diet was altered to one chiefly consisting of fish, fowl, and milk, with a moderate amount of starch food, and three pints of distilled water daily, a rapid change occurred. The oxalates, blood, and albumen vanished, the urea diminished, and the quantity of the water increased. An alkaline draught night and morning was given for a short time, and the patient became perfectly well. Similar instances of hæmaturia and oxalates were given in the *Lancet* a few years ago, in which the patients had eaten freely of rhubarb; and both tea, unripe fruits, and asparagus have occasionally produced the same symptoms. Indeed, Dunlop's argument that oxalates are merely ingested with the food is supported by the fact that on a milk diet even the small normal amount of oxalates in the urine vanishes entirely.

The deposition of phosphates is almost as obscure as the production of oxalates. J. B. Nichols gives[9] a very full discussion and bibliography of the different views which have been held by physiologists on the true explanation of the precipitation of phosphates caused by heating certain urines. In estimating the various theories he concludes that this precipitation may be due to chemical alteration by heat, less soluble salts being formed; the process being reversed on cooling, when the salts redissolve. Besides this change of the salts the original compound is probably less soluble in hot than in cold media, and is therefore, if unchanged in chemical composition, thrown down on heating. Finally, the expulsion of $CO_2$ and loss of acidity caused by heating may explain in part those cases where turbidity remains after cooling.

The occurrence of this precipitation does not depend alone on the amount of the phosphates present, but also on the acid phosphates and chlorides which hold them in solution. Hence it may occur either in excess of phosphates with high acidity, or when the phosphates are scanty but the acidity very low, and, in short, has little clinical significance.

[1] Am. J. Med. Sc., Nov. [2] Les. Nouv. Remèdes, July 24. [3] Clin. Jour., Aug. 25. [4] B. Med. Jour., Nov. 6. [5] Med. Chron., June. [6] Clin. Jour., Sept. 1. [7] Internat. Med. Mag., Oct. [8] Birmingham Med. Rev., Sept. [9] Med. Record, Oct. 30.

